# Which ovarian reserve marker relates to embryo quality on day 3 and blastocyst; age, AFC, AMH?

**DOI:** 10.5935/1518-0557.20200060

**Published:** 2021

**Authors:** Juliano Brum Scheffer, Rafaela Friche de Carvalho, Ana Paula de Souza Aguiar, Iole Joana Moreira Machado, Juliana Baumgratz Franca, Daniel Mendez Lozano, Renato Fanchin

**Affiliations:** 1 IBRRA - Brazilian Institute of Assisted Reproduction, Belo Horizonte, Brazil; 2 School of Medicine, Tecnologico de Monterrey and Center for Reproductive Medicine CREASIS, San Pedro Monterrey, Mexico; 3 Professeur des Universites - Praticien Hospitalier en Medecine de la Reproduction, France; Hopital Foch, France

**Keywords:** AMH, age, antral follicle count, ovarian reserve, embryo quality

## Abstract

**Objective:**

The aim of the present prospective study was to evaluate which ovarian reserve marker would be more reliable as the quality of the A + B embryos (day 3 and blastocyst).

**Methods:**

We ran a prospective study with 124 infertile women, aged 24-48 years, from 2017 to 2018. The patients were divided into 3 groups according to age and the subgroups were compared for AMH, AFC, number of A+B embryos. New division of the 3 groups was performed based on the AMH, and the subgroups were compared for age, AFC and number of A+B embryos. Finally, we divided the patients into 3 groups, based on the AFC, and we compared the subgroups for age, AMH and number of A+B embryos. *P*<0.05 was considered statistically significant.

**Results:**

When the 124 patients were divided according to age, we found a significant fall in an A+B embryo quality (day3; blastocyst) after 35 years (*p*<0.038; *p*<0.035), and more severely after 37 years (*p*<0.032; *p*<0.027). When the 124 patients were divided according to AMH, there was a significant fall in A+B embryo quality (day 3; blastocyst), with AMH<1ng/ml (*p*<0.023; *p*<0.021). When the 124 patients were divided according to AFC, there was a significant fall in A+B embryo quality (day 3; blastocyst) with AFC<7 (*p*<0.025; *p*<0.023). These markers had significant associations with embryo quality (*p*<0.005).

**Conclusion:**

Age, AFC and AMH have significant associations with A +B embryo quality on day 3 and blastocyst.

## INTRODUCTION

Fertility rates start to decrease in women older than 35 years of age, due to a decrease in the number of normal oocytes available. This process is a consequence of oocyte atresia and, although this normally happens to happen to all women, there is no certainty in predicting the rate of decay. This decay is age-related, where there is a decrease in ovarian quality, number of oocytes and markers of ovarian activity through a gradual increase in circulating FSH, and a decrease in circulating anti-Müllerian hormone (AMH), and antral follicle count (AFC).

The effects of female age on fertility was found in a classic report, where the percentage of women who did not use contraceptives remained childless, steadily after increasing according to their marriage age: 6% in the 20 to 24 age group; 9% in the 25 to 29 age group; 15% in 30-34 years old; 30% in 35-39 years old and 64% in 40-44 year-olds (Vigier *et al*., 1984).

Success rates in assisted reproductive technologies in 2001 show that the percentages of clinical pregnancies (ultrasound-visible gestational sac) that did not result in a live birth were 14% for women under 35 years of age; 19% for those 35 to 37 years of age; 25% for those 38 to 40 years old and 40% for those over 40 years old (CDC, 2003).

Age-related decay in female fertility and increased risk of miscarriage are largely attributed to oocyte abnormalities. The meiotic spindle in the oocytes of elderly women regularly exhibits abnormalities in chromosomal alignment and microtubular matrix composition (di Clemente *et al*., 1992). Higher rates of single chromatid abnormalities in oocytes (Weenen *et al*., 2004), aneuploidy in preimplantation embryos (Durlinger *et al*., 2002) and ongoing pregnancies may be found in older women. The rate of aneuploidy is a major cause of increased miscarriage and decreased live birth rates in women of advanced reproductive age.

Ovarian reserve assessment has recently been the focus of many clinical studies (Guibourdenche *et al*., 2003; Rajpert-De Meyts *et al*., 1999; La Marca *et al*., 2005; de Vet *et al*., 2002; Fanchin *et al*., 2003). Thus, anti-Müllerian hormone (AMH), also called Müller inhibitory substance, is a dimeric glycoprotein that belongs to the transforming growth factor-b (TGF-b) superfamily, such as activins and inhibins, being produced exclusively in the gonads. In females, AMH is synthesized by granular cells (GC) around the preantral and small antral follicles (Weenen *et al.,* 2004; Durlinger *et al*., 2002). AMH is almost undetectable in serum at birth, and it can reach higher levels after puberty (Guibourdenche *et al*., 2003; Rajpert-De Meyts *et al*., 1999), although it then decreases with advancing age, where it becomes again undetectable at menopause (La Marca *et al*., 2005). Although the physiological roles and mechanisms involved in AMH regulation are not yet precisely determined, recent studies have pointed to this hormone as a viable marker for examining ovarian activity.

Basal AMH, which can be determined prior to stimulation (usually on day 3 of the cycle), was considered a better marker for assessing decreased ovarian reserve when compared with the classic parameters: increased follicle stimulating hormone (FSH), decreased inhibin B, or antral follicle count (de Vet *et al*., 2002; Fanchin *et al*., 2003; 2005; Muttukrishna *et al*., 2005; Tremellen *et al*., 2005; Hazout, 2006). In addition, AMH is inversely correlatedwith age andbaseline FSH values; and it is directly correlated with AFC (Piltonen *et al*., 2005).

According to assisted reproduction technology (ART), AMH serum, AFC, and age also prove to be the most reliable hormonal marker of ovarian response to gonadotropin-controlled ovarian hyperstimulation (COH), rather than baseline FSH, estradiol, and inhibin B levels (Anckaert *et al*., 2012; Hazout *et al*., 2004; Muttukrishna *et al*., 2004; Nardo *et al*., 2009; Peñarrubia *et al*., 2005; Seifer *et al*., 2002). However, AMH, AFC and age are not a good predictor of embryo quality or pregnancy in controlled ovarian stimulation cycles. Thus, these markers are suggested to be only quantitative and not qualitative for the ovarian reserve (Broer *et al.*, 2011; Nelson *et al*., 2007; Smeenk *et al*., 2007). Age can, nevertheless, be a good predictor of embryo quality (Scheffer *et al*., 2017).

Currently, we check IVF cycle success by embryo quality, where embryonic development is assessed by parameters associated with their morphological appearance, or markers, which can thus determine embryo health and quality (Puissant *et al*., 1987; Gardner *et al*., 2000).

The goal of the present prospective study was to evaluate which ovarian reserve marker would be most reliable regarding the quality of A + B day 3 embryos and blastocyst; and demonstrate whether age, AFC and AMH are markers of quality and not just oocyte quantity.

## MATERIAL AND METHODS

### Subjects

We ran a prospective study involving 124 infertile women, aged 24-48 years, undergoing routine exploration during an unstimulated cycle that preceded ART at our center, from May 2017 to October 2018. All patients met the following inclusion criteria: i) both ovaries present, ii) no current or past diseases affecting ovaries, gonadotropin or sex steroid secretion, clearance, or excretion, iii) no current hormone therapy, iv) adequate visualization of ovaries at transvaginal ultrasound scans, and v) total number of small antral follicles (3-12 mm in diameter) between 1 and 32 follicles, including both ovaries, and vi) no male infertility. All patients signed an informed consent form for this analysis.

### Protocol

We gave the patients leuprolide acetate (Lupron, Abbott, France), and started the GnRH-agonist at a dose of 2,0 mg per day during the midluteal phase, with approximately a 5-day overlap with the contraceptive (Diane 35, Schering, Brasil). We monitored their pituitary down-regulation, and patients with adequate pituitary desensitization started their recombinant FSH regime (Gonal-F; Merck-Serono Pharmaceuticals, Italy), and reduced the GnRH-agonist dose to 1,0 mg per day. We started the FSH with dosages between 150 and 300 IU daily for 4 days, with or without human menopausal gonadotropin (HMG) (Menopur; Ferring Pharmaceuticals, Germany). Thereafter, we adjusted the FSH dose individually, according to the estradiol (E2) response and vaginal ultrasound findings.

When two follicles reached ≥16 to 18 mm, 250 mg, we administered recombinant human Chorionic Gonadotropin (Ovidrel, Merck-Serono Pharmaceuticals, Italy) and retrieved oocytes 35 to 36 hours later.

We routinely performed intracytoplasmic sperm Injection (ICSI) in all the fertilization procedures. Fertilization was evident when we spotted two pronuclei. The embryos were cultured until the day of transfer (blastocyst) in IVF Global® media (Life Global, Canada), supplemented with 10 % synthetic serum substitute (SSS), and we graded the embryos on day 3 using the Veeck’s (Veeck, 1999) criteria, and the blastocyst by Gardner’s grading scale (Gardner *et al.,* 2000).

The same embryologist performed all embryology and embryo scoring in this study. We classified the embryos on day 3 and blastocyst (day 5).

### Hormonal Measurements and Ultrasound Scans

On day 3 of the cycle, preceding COH, we submitted the women to blood sampling by venipuncture for serum AMH, and FSH measurement, and a transvaginal ovarian ultrasound scan for follicle measurement.

We determined AMH and FSH serum levels using an automated multianalysis system, with chemiluminescence detection (ACS-180; Bayer Diagnostics, Puteaux, France). Serum AMH levels were determined using a second generation enzyme-linked immunosorbent assay. Intra- and inter- assay variation coefficients (VC) were < 6 and <10% respectively, with lower detection limits of 0.13 ng/ml and linearity up to 21 ng/ml for AMH. The functional sensitivity for FSH was 0.1mIU/ml, and intra-assay and interassay CV were 3% and 5%, respectively.

Ultrasound scans were performed using a 3.7-9.3 MHz multifrequency transvaginal probe (RIC5-9H; General Electric Medical Systems, Paris, France) by a single operator who was blinded as to the results of the hormone assays. The ultrasound examination aimed at evaluating the number and size of small antral follicles. Follicles measuring 3-12 mm in mean diameter (mean of two orthogonal diameters) in both ovaries were considered. To optimize the ovarian follicular assessment reliability, the ultrasound scanner was equipped with a tissue harmonic imaging system, which allowed improved image resolution and adequate recognition of follicular borders. Intra-analysis CV for follicular and ovarian measurements were <5%, and their lower limit of detection was 0.1 mm. In an effort to evaluate the bulk of granulosa cells in both ovaries, we calculated the mean follicle diameter (cumulative follicle diameter divided by the number of follicles measuring 3-12 mm in diameter in both ovaries) and the largest follicle diameter.

### Groups and Subgroups

The patients were divided into 3 groups according to age; <35 years (62 patients), 35-37 years (31 patients) and >37 years (31 patients). After this division, the groups were compared for AMH, AFC and number of A+B embryos (day 3 and blastocyst). Thereafter, we divided the 3 groups again, based on the AMH; <1ng/ml (32 patients); 1-2 ng/ml (32 patients); >2 ng/ml (60 patients) and we compared the groups for age, AFC and number of A+B embryos (day 3 and blastocyst).

Finally the patients were again divided into 3 groups based on the AFC; >7 (30 patients); 8-14 (34 patients); > 14 (60 patients) and the groups were compared for age, AMH, number of A+B embryos (day 3 and blastocyst). The criteria used in the group divisions and subgroups regarding age, AMH and AFC were based on the scientific literature (Bishop *et al*., 2017; Kim, 2017).

These divisions in groups based on the main ovarian reserve markers aimed at evaluating which marker would be more reliable as the quality of the A + B embryos (day 3 and blastocyst).

### Statistical Analysis

Descriptive parameters and patient characteristics were reported as mean SD or median (range), depending on the distribution. The Student’s t-test was performed for continuous variables; Wilcoxon and Pearson’s Test were used where appropriate for categorical variables. *p<* .05 was considered statistically significant.

### Ethical approval and consent to participate

Written informed consent was obtained from all participants before inclusion. The study was approved by the IBRRA Ethical Committee. Our patients signed an informed consent form for this analysis. Because the present study was merely observational and included only data from routine measurements, it did not require previous submission to our institutional review board.

## RESULTS

Overall, at the time of this investigation, patients had a mean age of 34.28±4.02 years old; BMI of 24.92±3.14kg/m^2^; and length of infertility of 3.44±2.44 years. On cycle day 3, the serum AMH level was 2.69±2.52ng/ml and the serum FSH level was 12.68±9.26mUI/ml. At baseline, women had 12.53±5.34 antral follicles. Tables 1, 2 and 3 demonstrate the characteristics of each subgroup.

**Table 1:** Characteristics of Subgroups by Age (years)

Age Subgroups	BMI	AMHd3	AFCd3	FSH d3	Total FSH dose	
<35 years	24.26± .91	3.34±2.31	14.78±4.55	9.36±4.10	1,539±443.15	
≥35-≤37 years	25.59±3.36	2.28±3.19	10.29±5.43	16.26±10.80	2,190±788.21	
>37 years	25.62±3.24	1.71±1.70	10.17±4.95	15.89±12.84	2,025±426.33	
Age Subgroups	Stimulation duration	Foll total	MII	Total Embryos	A+B Embryos (day3)	A+B Embryos (Blastocyst)
<35 years	10.28±2.00	13.10±5.37	7.68±3.92	5.23±2.94	2.58±2.05	2.10±0.95
≥35 -≤ 37 years	10.33±1.49	10.00±4.56	7.29±4.43	5.29±3.48	3.29±2.74	2.09±0.88
>37 years	10.50±1.62	8.72±5.07	4.56±3.24	3.22±2.60	1.39±1.65	1.20±0.70

**Table 2:** Characteristics of AMH subgroups (ng/ml)

AMH Subgroups	BMI	Age	AFCd3	FSH d3	Total dose of FSH	
0 - 1 ng/ml	25.91±2.83	36.77±3.29	6.50±2.54	23.23±11.79	2.600±510.03	
1.1 - 2 ng/ml	23.88±2.59	33.83±3.94	10.72±2.05	11.28±2.05	1.675±297.61	
> 2 ng/ml	24.84±3.43	33.08±3.88	16.77±3.47	7.39±1.77	1.453±323.02	
AMH Subgroups	Stimulation duration	Foll total	MII	Total Embryos	A+B Embryos	A+B Embryos (Blastocyst)
0 - 1 ng/ml	10.27±1.35	5.96±3.05	4.59±2.59	3.23±1.9	1.64±1.62	1.02±0.83
1.1 - 2 ng/ml	10.11±1.41	10.39±4.16	6.44±4.15	4.50±3.45	2.06±1.86	1.98±0.99
> 2 ng/ml	10.49±2.13	14.54±4.41	8.33±4.18	5.80±3.16	3.18±2.53	2.07±1.00

**Table 3:** Characteristics of AFC subgroups (n)

AFC Subgroups	BMI	Age	AMH	FSH d3	Total dose of Medicines	
0 -7	25.00±2.50	36.93±3.53	0.61±0.37	25.87±13.42	2.671±419.08	
8 -14	24.72±3.08	34.24±4.09	1.74±0.75	11.31±4.17	1.803±508.34	
> 14	25.15±3.60	32.85±3.53	5.14±2.87	7.25±1.61	1.380±284.30	
AFC Subgroups	Stimulation duration	Foll total	MII	Total Embryos	A+B Embryos	A+B Embryos (Blastocyst)
0 -7	10.20±1.47	5.20±3.53	4.73±2.89	3.33±2.19	1.60±1.80	1.93±0.56
8 -14	10.19±1.33	10.49±3.88	6.46±3.81	4.57±3.02	2.32±1.92	2.10±0.76
>14	10.63±2.39	15.52±4.39	8.59±4.40	5.89±3.33	3.22±2.71	2.70±1.80

When the 124 patients were divided according to age, there was a significant fall in AMH and AFC among those older than 35 years (*p*<0.006; *p*<0.003), and it was more severe after 37 years of age (*p*<0.003; *p*<0.002). And the fall in A+B embryo quality (day 3; blastocyst, respectively) was significant after 35 years (*p*<0.038; *p*<0.035) and even more severe after 37 years (*p*<0.032; *p*<0.027).

When the 124 patients were divided according to AMH, there was a significantly negative correlation with age (*p*<0.05), and this was more intense with AMH <1 ng/ml (*p*< 0.001); as well as a significantly positive relationship with AFC (*p*<0.05) and more intense with AMH>2 ng/ml (*p*<0.001). And the fall in A+B embryo quality (day 3; blastocyst, respectively) was significant with AMH<1 ng/ml (*p*<0.023; *p*<0.021).

When the 124 patients were divided according to AFC, there was a significantly negative correlation with age (*p*<0.05), and this was more intense with AFC<7ng/ml (*p*<0.001); as well as a significantly positive relationship with AMH (*p*<0.05), and even more intense with AFC>14 (*p*<0.006). And the fall in A+B embryo quality (day 3; blastocyst, respectively) was significant with AFC<7 (*p*<0.025; *p*<0.023).

Table 4 shows that markers, age, AFC and AMH had significant associations with A+B embryo quality on day 3 and that the strength of significance was higher with AMH (*p=*0.307; *p*<0.006) and AFC (*p=*0.310; *p*<0.005) than age (*p=* -0.137; *p*<0,023).

**Table 4:** Associations between age, AMH and AFC with day 3 Embryo-Quality (Pearson's Test)

	*p*	*p*-value
Age x Embryos A+B	-0.137	0.023
AMH x Embryos A+B	0.307	0.006
AFC x Embryos A+ B	0.310	0.005

However, Table 5 shows that the strength of significance was equal between AMH (p=0.290; *p*<0.005), AFC (p=0.295; *p*<0.005), and age (*p*= -0.28; *p*<0.005) with blastocyst quality.

**Table 5:** Relationship between markers age, AMH and AFC with Embryos Blastocyst Quality (Pearson's Test)

	*p*	*p*-value
Age x Embryos A+B	-0.280	0.005
AMH x Embryos A+B	0.290	0.005
AFC x Embryos A+ B	0.295	0.005

## DISCUSSION

This study validated the association between clinical measures often used in ovarian reserve and embryo quality on the third day and in the blastocyst stage. There was an association between basal AMH, age and AFC with embryo quality. Thus, they may contribute as a possible explanation to the results of some studies that show the associations of markers with the probability of pregnancy, since embryo quality is fundamental for clinical success. There are suggestions that these markers are quantitative and qualitative vis-à-vis the ovarian reserve. Although ovarian reserve markers demonstrate clinical importance in assisted reproduction treatments, they may indicate not only clinical but embryo prognosis as well.

Our group demonstrated that female age is a predictive marker of the number of oocytes collected, number of oocytes in MII and embryo quality (Scheffer *et al*., 2017). This relationship between age and embryo quality is due to the oocyte quality that will then influence embryo quality. Women transfer half of the chromosomal complement to the embryo, although the maternal and paternal genomes are not symmetrical and equal in their influence on the embryo. Oocytes unfortunately show a drop in quality with age, due to genetic changes. The incidence of non-disjunction and early chromatid separation correlated to maternal aging. Disturbance in sister chromatid cohesion might be a causal mechanism predisposing to premature chromatid separation and subsequently to nondisjunction in female meiosis. In addition, the asymmetry of female meiosis division could favor a nonrandom meiotic segregation of chromosomes and chromatids.

In some studies, there was an age-association with the expression of certain genes and proteins involved in mitochondrial function. Mitochondria play a role in cellular energy metabolism, homeostasis and cell death, and are directly involved in oogenesis and folliculogenesis. With age, there is an increase in damage to mitochondrial DNA. Mitochondrial mutations in follicular cells are observed in oocytes of older women, causing a decrease in the quality of these oocytes (McReynolds *et al.*, 2012).

Age correlates with embryo quality and also with AMH and AFC. This paper reports that these two ovarian reserve markers may also be associated with embryo quality. We assessed the correlation of these markers with embryo quality on day 3, where the main genetic expression is maternal, while in the blastocyst stage it is more embryonic, that is; it has both paternal and maternal performance. This assessment is important because these markers reflect the most advanced embryonic development, of which maternal and paternal gene expression is most significant. Although there was no case of male infertility, it is practical to perform ICSI in all cases.

Oxidative stress may affect embryo quality (Agarwal *et al*., 2005), although even morphologically normal embryos may have an abnormal number of chromosomes and low pregnancy rates. However, the possibility that the main factor in the etiology of female infertility is associated with age is that the decline in oocyte quality is associated with factors such as chromosomal aneuploidy and mitochondrial dysfunction (Twisk *et al*., 2006; Cheng *et al*., 2009; Eichenlaub-Ritter *et al*., 2011). However, the underlying mechanisms are still inconclusive.

The objective of the study was to assess embryo quality, so no pregnancy rate was analyzed. This is yet to be evaluated by the authors. It is important to highlight some limitations present in the study, such as the small sample size, which may limit the ability to demonstrate the additional value of these markers with embryo quality. In addition, there are many published embryo scoring systems (Desai *et al*., 2000; Steer *et al*., 1992; Hoover *et al*., 1995; Rienzi *et al*., 2002; Fisch *et al*., 2001). Despite the systematic approach of such scoring systems to compare and contrast embryos, embryo morphology and assigning of a grade is, by default and design, a subjective process subject to interobserver and intraobserver variability - although in our case all embryos were evaluated by the same embryologist.

Initially the pregnancy rate, ongoing pregnancy rate or live birth rates were not the primary objective of this study. But the authors are finishing other studies that will be added to these rates as a primary objective, thus bringing new knowledge about these markers in the future. Despite possible biases, this study is important in that it demonstrates that these markers can reflect both the quantity and quality of the ovarian reserve, and encourage further studies on this topic. Moreover, due to the limitations of physicians in their countries, especially in poor or developing countries, in evaluating all three markers, this study also ensures that the evaluation of at least one marker has already enabled physicians to have better embryonic prognoses.

Embryo quality assessment can also be performed using invasive methods, such as PGT-A, and non-invasive methods such as Time Lapse. However, both methodologies have flaws due to their subjectivities, damage to the embryo and questionable results. Because some centers are unable to use these methodologies, other evaluation methods should also be analyzed according to the objective of this study.

Therefore, more studies can improve the accuracy and interpretation of current ovarian reserve markers relating to embryonic quality and clinical pregnancy rates.

## CONCLUSION

In summary, we demonstrated that commonly used clinical markers of ovarian reserve are reflective of the true ovarian reserve, and AMH, AFC and age are markers of embryo quality; therefore, they are markers of ovarian reserve quantity and quality.
